# Mechanisms and therapeutics of insulin signaling transduction genes in diabetic cardiomyopathy: a comprehensive updated review

**DOI:** 10.3389/fendo.2025.1589695

**Published:** 2025-07-17

**Authors:** Yufeng He, Xi Yang, Xinghui He, Guoshuang Wang, Chuang Li, Ping Yuan, Chunhong Li

**Affiliations:** ^1^ Department of Cardiology, The Affiliated Hospital of Southwest Medical University, Luzhou, Sichuan, China; ^2^ Department of Pharmaceutical Sciences, School of Pharmacy, Southwest Medical University, Luzhou, Sichuan, China; ^3^ Department of Neurology, The Affiliated Hospital of Southwest Medical University, Luzhou, Sichuan, China

**Keywords:** diabetic cardiomyopathy, heart failure, insulin signaling, gene, treatment

## Abstract

Diabetic cardiomyopathy (DCM), a complication of type 2 diabetes mellitus (T2DM), is closely associated with key genes in the insulin signaling pathway. Insulin regulates cellular metabolism and growth under normal conditions by activating downstream signaling pathways through its receptors. Nonetheless, insulin resistance, which compromises the insulin signaling pathway and impairs cardiovascular system performance, is common in individuals with T2DM. The key insulin signaling genes include IRS1, IRS2, PIK3R1, and GLUT4 play important roles in insulin receptor signaling, PI3K complex assembly, and glucose transport, respectively. Mutations or abnormal expression of these genes may lead to disorders in the insulin signaling pathway, affecting the normal regulation of glucose metabolism and impairment of myocardial function, thereby promoting the development of DCM. This review delves into the specific roles of these genes in the pathogenic mechanisms and treatment of DCM, with the aim of providing scientific evidence and guidance for future research endeavors.

## 1 Introduction

The most commonly accepted definition of diabetic cardiomyopathy (DCM) is cardiac dysfunction in patients with diabetes mellitus in the absence of other cardiovascular diseases (e.g., coronary artery disease, uncontrolled hypertension, severe valvular heart disease, and congenital heart disease) (1). This type of disease is primarily caused by changes in the function and structure of the heart due to metabolic abnormalities in the diabetic body, such as hyperglycemia (2). The initial phase is characterized by concentric left ventricular hypertrophy, increased myocardial stiffness, elevated atrial filling pressures, and impaired diastolic function. Seferovic et al. proposed that DCM may manifest as two distinct phenotypes rather than as a series of consecutive stages of the same disease: either restrictive/heart failure with preserved ejection fraction (HFpEF) or dilated/heart failure with reduced ejection fraction (HFrEF). The distinction between these two categories may indicate disparate prognoses and treatment pathways, thereby warranting further investigation, as there are several effective pharmacological treatments for HFrEF in this context, but evidence remains limited (3).

A definitive diagnosis of DCM remains difficult due to the lack of uniform diagnostic criteria and consistency in the definition of the disease. In recent years, the number of novel drugs for the treatment of coronary artery disease (CAD) and heart failure (HF) has notably increased. The sodium–glucose cotransporter protein 2 inhibitor (SGLT2i) class of drugs has been shown to improve cardiac function in diabetic patients with DCM. Additionally, SGLT2i treatment has demonstrated considerable ameliorative effects on several physiological functions, including the promotion of metabolic homeostasis, improvement of microcirculatory hemodynamics, enhancement of mitochondrial energy metabolism function, reduction of fibrosis in the myocardium and other tissues, decrease of oxidative stress-induced cellular damage, and alleviation of the protein misfolding problem caused by endoplasmic reticulum (ER) stress. Furthermore, it inhibits programmed cell death, regulates autophagy to maintain intracellular stability, and optimizes the composition and function of the intestinal flora, thereby achieving a comprehensive multi-system protective effect (4). However, the exact targets and signaling pathways of SGLT2i in clinical therapy are still not fully understood, and effective treatments for myocardial lipotoxicity in DCM have yet to be established (5, 6). Understanding the link between diabetes mellitus and DCM and exploring possible effective preventive and therapeutic strategies are urgently important.

## 2 Pathophysiological feature of DCM

**Figure 1:**
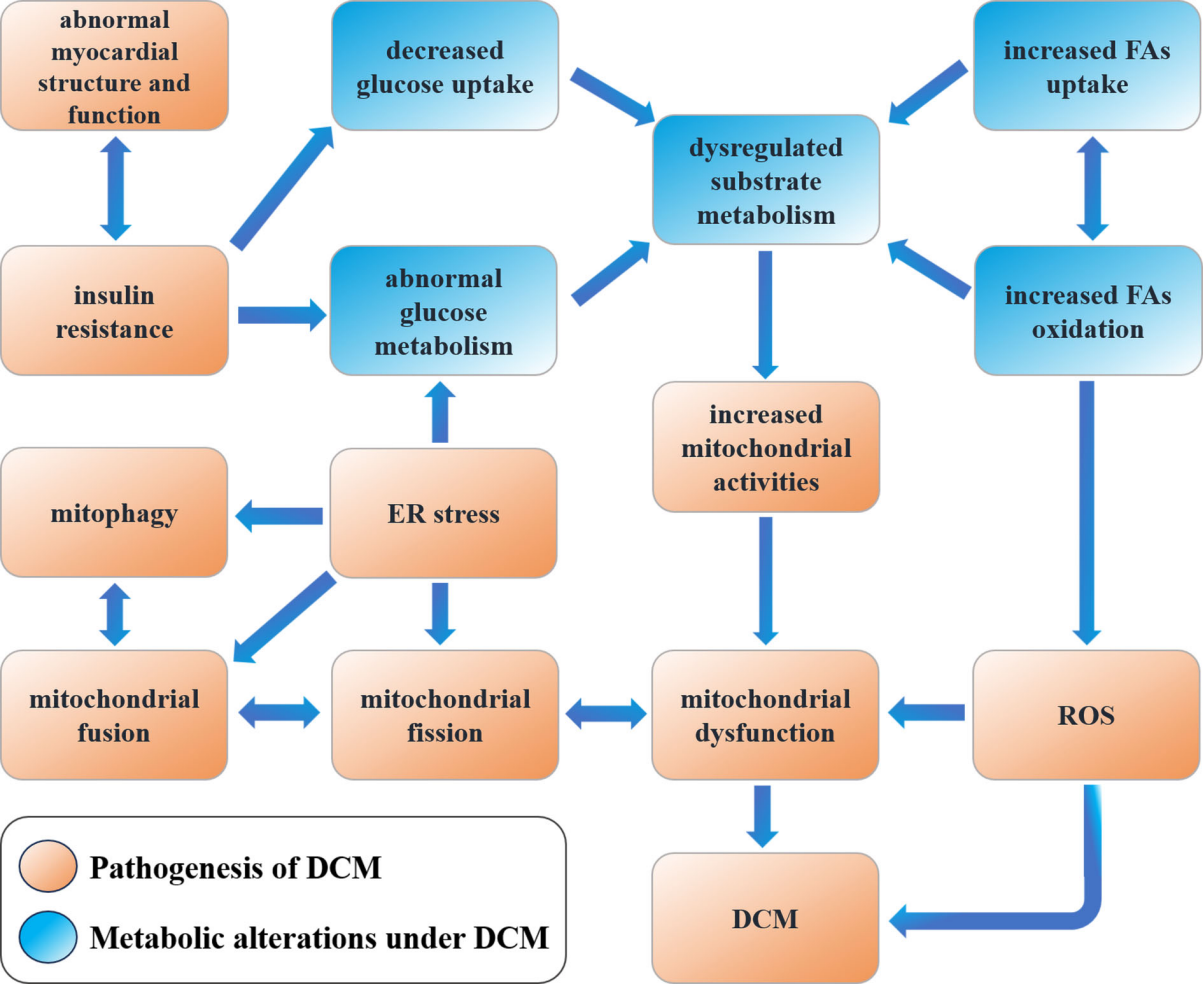
Metabolic alterations and pathophysiological processes of DCM.

DCM presents complex pathophysiologic features clinically and is closely associated with the development of cardiac disease. Long-term chronic hyperglycemic states can result in pathological changes in cardiac structure and function due to cardiac metabolic disorders, abnormal mitochondrial function, inflammation, oxidative stress, and myocardial fibrosis, leading to alterations in cardiomyocyte structure (7). Under physiological conditions, both glucose and fatty acids can serve as the primary energy source for the heart, and their amounts can be dynamically regulated, providing flexibility in myocardial energy metabolism (8). Both differentiation cluster 36 (CD36) and fatty acid transferase (FAT) are involved in mediating fatty acid uptake in the myocardium, whereas glucose uptake is mediated by GLUT4-mediated insulin-stimulated glucose transport (9, 10). Increased blood glucose concentrations elevate plasma insulin levels and myocardial insulin signaling, further promoting GLUT4 and CD36 translocation to cardiomyocyte sarcoplasm to provide myocardial energy. However, this translocation is inhibited in the context of insulin resistance in T2DM patients, resulting in an increased proportion of free fatty acid oxidation (FAO) in myocardial metabolic substrates and reduced cardiac efficiency (11). The excessive accumulation of fatty acids in cardiac tissues, coupled with associated lipotoxicity, impairs insulin signaling and reduces physiological autophagy in cardiomyocytes. This leads to morphological and structural alterations in the myocardium, as well as a reduction in the efficiency of the myofibrillar response to electrical stimuli. Furthermore, it increases myocardial oxygen consumption, ultimately impairing myocardial function (12). Additionally, chronic and persistent hyperglycemia has been demonstrated to impair cardiac structure and function through the generation of oxidative stress by producing increased amounts of reactive oxygen species (ROS), which inhibit the activity of glyceraldehyde-3-phosphate dehydrogenase (G3PDH), resulting in DNA damage within cardiomyocytes and the formation of advanced glycation end-products. These factors stimulate collagen expression and accumulation, as well as collagen cross-linking. DCM can lead to increased myocardial fibrosis and decreased compliance (13). Decreased ATP production and increased ROS production affect mitochondrial structure and function in this disease. Decreased ATP is usually accompanied by impaired mitochondrial electron transport chain (ETC) function, leading to decreased efficiency of the mitochondrial respiratory chain. Prolonged production of ATP can trigger mitochondrial autophagy, which may further reduce ATP production and increase ROS. Increased ROS leads to altered permeability of the mitochondrial membrane, affecting the normal ionic balance and ATP synthesis inside and outside the mitochondrial membrane. In addition, various other mechanisms, including the promotion of mitochondrial DNA (mtDNA) mutations, affect the structure and function of mitochondria, leading to cellular dysfunction and cell death (14). **Figure 1** demonstrates the metabolic alterations and pathophysiological processes in DCM.

At the subcellular level, chronic hyperglycemia disrupts mitochondrial dynamics via ERK/Drp1 signaling, leading to excessive mitochondrial fission and impaired respiratory chain function (15, 16). This results in reduced ATP synthesis (from 30% to 50% loss in diabetic hearts) and elevated ROS production, which directly damages mtDNA and activates apoptosis through caspase-3 pathways (17, 18). Morphologically, cardiomyocytes exhibit sarcomere disarray, lipid droplet accumulation, and interstitial collagen deposition, culminating in both diastolic and systolic dysfunction (19–21). Notably, persistent hyperglycemia promotes advanced glycation end-products (AGEs) formation, which cross-links collagen fibers and increases myocardial stiffness compared to non-diabetic controls, as evidenced by elevated left ventricular end-diastolic pressure (LVEDP) in echocardiography (22–24). The transition from compensatory hypertrophy to overt heart failure involves a shift in metabolic substrate utilization (25). Insulin resistance suppresses GLUT4-mediated glucose uptake, forcing cardiomyocytes to rely on FAO, which accounts for 50-70% of ATP production in early DCM (26, 27). However, excessive FAO generates lipotoxic intermediates (e.g., ceramides) that further impair insulin signaling and induce ER stress, creating a vicious cycle (28, 29).

At the molecular level, hyperglycemia activates pro-fibrotic pathways including TGF-β/Smad3 and Wnt/β-catenin signaling (30, 31). TGF-β1 stimulates cardiac fibroblasts to secrete collagen I/III, increasing extracellular matrix (ECM) volume, while Wnt signaling upregulates fibrosis-related genes like WISP-1 (32, 33). Concurrently, inflammatory pathways such as NLRP3 inflammasome and nuclear factor-κB (NF-κB) are activated, amplifying IL-6 and TNF-α production, which directly impair cardiomyocyte contractility by reducing SERCA2a activity and calcium handling efficiency (34, 35).

In studies of patients with DCM, it was found that the serum oxidative stress markers were significantly higher than those of healthy control groups (36). These oxidative stressors can lead to lipid peroxidation, protein oxidation, and DNA damage in the cell membrane, which in turn can affect cardiomyocyte function and lead to apoptosis suggesting that a chronic inflammatory state often exists in patients with DCM (37). Chronic inflammation not only exacerbates oxidative stress through direct ROS production but also maintains and amplifies the inflammatory response through multiple mechanisms (38). Sex hormones exert further regulatory effects on these pathways. Estrogen confers cardioprotective benefits by promoting mitochondrial biogenesis and mitigating oxidative stress (39, 40). In contrast, testosterone may promote cardiac fibrosis through the TGF-β signaling pathway (41). These hormonal interactions may, in part, account for the higher prevalence of myocardial stiffness observed in postmenopausal women with DCM (42). In addition, traditional cardiac risk factors such as dyslipidemia and hypertension are often more severe in patients with DCM. Hyperglycemia and hyperinsulinism can promote the generation of oxidative stress, further leading to the impairment of endothelial cell function. In turn, endothelial dysfunction can further exacerbate the state of metabolic derangement and oxidative stress, resulting in a vicious cycle (43).

The mechanism of action of many drugs is based on the modulation of the aforementioned pathophysiological features of DCM to treat the disease. For example, the natural compound phloridzin has anti-inflammatory and antioxidant properties (44). It inhibits myocardial fibrosis and exerts antioxidant effects in high glucose-stimulated animal models and an *in vitro* H9C2 cell model. Cardamonin, a flavonoid found in Alpinia, has a profound protective effect on DCM in a mouse model of type 2 diabetes mellitus (T2DM) by inhibiting macrophage M1 polarization to prevent cardiomyocyte damage (45). While rodent models have been instrumental in elucidating insulin signaling pathways and metabolic dysregulation in DCM, critical limitations must be acknowledged when translating findings to human pathophysiology. First, the accelerated metabolic rates and compressed disease timelines in rodents (e.g., DCM developing within months versus years in humans) may distort the comparability of oxidative stress and fibrotic progression. Second, hormonal imbalances, particularly the interplay between estrogen and testosterone, introduce confounding variables. Rodent estrous cycles differ fundamentally from human postmenopausal hormonal environments, potentially exaggerating estrogen’s cardioprotective effects observed in murine studies. Similarly, human sex-specific comorbidities (e.g., androgen-driven fibrosis in males) are poorly replicated in standard rodent models (46). Third, the absence of polypharmacy and multimorbidity in experimental designs (e.g., concurrent hypertension or atherosclerosis) oversimplifies the clinical reality of DCM. Most rodent models rely on single-gene modifications or isolated metabolic insults, neglecting synergistic interactions between hyperglycemia, hypertension, and dyslipidemia. Future studies should integrate multi-system approaches and humanized models to bridge these translational gaps.

In addition, nanotherapeutics play a considerable role in modulating the pathophysiological profile of DCM. Nanotherapeutics can provide effective delivery vehicles for therapeutics such as drugs, plasmids, peptides, and proteins, achieving high solubility, good bioavailability, low toxicity, and non-immunogenicity. In patients with T2DM, impaired insulin signaling leads to the development of insulin resistance and abnormal glycemic control. Studying the role of genes related to insulin signaling in DCM will have a positive effect on the future search for new therapeutic targets and could aid in the treatment of this disease. To this end, this review summarizes studies from the last 20 years and provides an in-depth understanding of the pathogenic mechanisms of DCM and directions for exploring new therapeutic strategies. In addition, we will also discuss the role of nanotherapeutics for DCM, which has gained increasing attention in recent years.

## 3 Insulin signaling and metabolic regulation in the myocardium

### 3.1 Insulin signaling transduction genes in DCM

The heart, as a muscle pump in the body that is undergoing contraction and diastole at all times, requires large amounts of ATP to maintain muscle contraction and diastole and to energize the ion pump (e.g., sarcoplasmic reticulum Ca²^+^-ATPase/SERCA and Na^+^/K^+^-ATPase) (47, 48). The term “energize” here specifically refers to the ATP-dependent activation of these ion pumps, which is facilitated by insulin signaling through the PI3K/Akt pathway (49). Insulin enhances glucose uptake via GLUT4, increasing intracellular glucose oxidation and ATP synthesis (50, 51). This ATP is then utilized by ion pumps to maintain transmembrane ion gradients critical for cardiac excitation-contraction coupling (52). Moreover, cardiac metabolism is largely controlled by the substrate supply. Thus, circulating insulin levels influence myocardial metabolism by regulating the concentration of myocardial metabolic substrates in the peripheral circulation and having a direct effect on the myocardium (53). The current research underscores the central role of insulin receptor substrates (IRS1/2) in maintaining cardiac homeostasis through the PI3K/Akt/Foxo1 signaling axis, which integrates metabolic regulation with survival mechanisms in cardiomyocytes (54). Therapeutic strategies targeting IRS stability (e.g., kinase inhibitors), Foxo1 activity, or mitochondrial biogenesis may restore metabolic plasticity and mitigate disease progression, highlighting the interplay between insulin signaling fidelity and cardiac resilience in diabetes (54).

**Figure 2:**
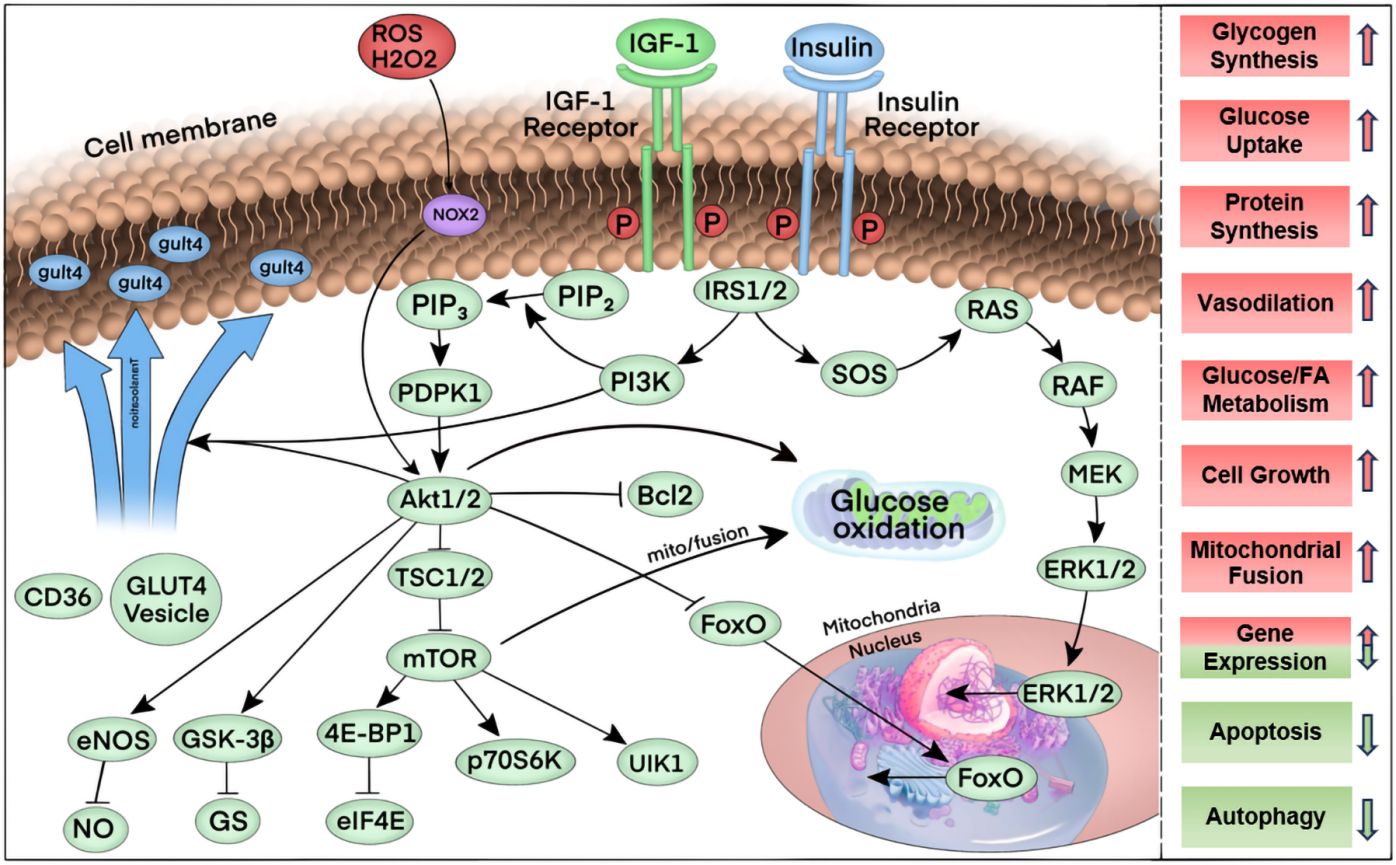
Summary of the key elements of the insulin signaling pathway. First, upon binding to its ligand, insulin and the IGF-1 receptor are phosphorylated, thereby increasing their tyrosine kinase activity. Tyrosine phosphorylation and activation of the docking proteins insulin receptor substrates 1 and 2 (IRS1/2) bind to the regulatory subunit of phosphatidylinositol 3-kinase (PI3K), which generates phosphatidylinositol 3,4,5-trisphosphate (PIP3) from phosphatidylinositol 4,5-bisphosphate (PIP2). The serine-threonine kinases phosphatidylinositol-dependent protein kinase 1 (PDPK1) and Akt1 or Akt2 bind to PIP3 via their PH structural domains. PDPK1 in turn further phosphorylates Akt1/2, which in turn phosphorylates multiple targets. Phosphorylation of these targets induces a variety of cellular responses: phosphorylation of Bcl2 inhibits apoptosis, phosphorylation of FOXO proteins promotes nuclear export, which inhibits the expression of FOXO-regulated transcripts that mediate autophagy and apoptosis. Tsc1/2 phosphorylation promotes the activation of mTOR, which is activated by the binding of eukaryotic translation initiation factor 4E-binding protein 1 (4E-BP1) to eukaryotic translation initiation factor 4E (eIF4E), ribosomal protein S6 kinase (p70S6K), and Unc-51-like autophagy activating kinase 1 complex (ULK1) pathways, thereby increasing mRNA translation, promoting protein synthesis, cell growth, mitochondrial fusion, and inhibiting autophagy, among other physiological effects. Phosphorylation of glycogen synthase kinase (GSK) removes glycogen synthase inhibition and promotes glycogen synthesis. Phosphorylation of endothelial nitric oxide synthase (eNOS) by Akt increases nitric oxide (NO) production to promote vasorelaxation. Phosphorylation of Akt partially mediates the translocation of vesicles containing the glucose transport protein GLUT4 through phosphorylation, leading to increased glucose transport after insertion into the plasma membrane.

Insulin receptors (IR) are abundantly expressed in cardiomyocytes, while the insulin-like growth factor 1 receptor (IGF1R) exhibits comparable expression levels. These two receptors share significant overlap in downstream signaling pathways within the myocardium (55). Similar to insulin signaling in other cell types, insulin binds specifically to the IR, whereas IGF-1 activates the IGF1R. Upon ligand binding, both receptors undergo autophosphorylation and subsequently recruit insulin receptor substrates IRS1 and IRS2. These substrates serve as signaling hubs for insulin signaling via protein kinase B (e.g., Akt) and phosphatidylinositol-3-kinase (PI3K), or members of the ERK signaling pathway, to the downstream pathway. Akt activation results in the phosphorylation of a number of downstream signaling intermediates, which drives the translocation of GLUT4 from the inside to the cellular membrane, increases the cellular uptake of glucose, and increases the activity of the target of rapamycin (mTOR). It also regulates the pathways of cell survival and glycogen metabolism and affects nitric oxide synthase isoform 3 (NOS3) to regulate cyclic guanosine monophosphate (cGMP) and NO production, including the regulation of Bcl-2-associated cell death promoters and members of the transcriptional regulator family, among others (49, 56, 57). The activation of insulin signaling plays a pivotal role in regulating a multitude of cellular processes within cardiomyocytes, encompassing metabolic functions, cell growth, cell survival, and the inhibition of apoptosis and autophagy. **Figure 2** shows in detail the flow of key elements of the insulin signaling pathway regulating multiple cellular processes.

Under normal physiological conditions, various proteins and effectors work together to regulate glucose metabolism and maintain glucose homeostasis. However, in patients with T2DM, the abnormal function of these proteins and effectors may lead to disturbances in insulin signaling, thereby affecting glucose metabolism and glycemic control. The latest research indicates that an imbalance in insulin signaling, whether insufficient or excessive, may contribute to the development of diabetic heart disease. This highlights the intricate relationship between the two. Compared with control mice, mice lacking insulin receptor (cytoplasmic insulin receptor knockout (CIRKO) expression in cardiac myocytes have more reduced left ventricular contractile function and greater interstitial fibrosis in the heart after pressure overload, suggesting a greater degree of left ventricular remodeling (58). In addition, in the diabetic model mice, fatty acid transport and utilization were increased in both the control and CIRKO groups but not in the nondiabetic CIRKO group. In contrast, myocardial metabolism, oxygen consumption, and the left ventricular ejection fraction were most severely impaired in the diabetic and CIRKO groups (59). These findings suggest that they synergistically contribute to the development and progression of DCM, which may be due to mitochondrial dysfunction and increased fatty acid utilization. For example, pressure overload-induced coronary artery dysfunction was reduced in CIRKO genotype mice, which presented elevated mRNA levels of vascular endothelial growth factor (VEGF), eNOS, and hypoxia-inducible factor-1α (HIF-1α). These parameters were elevated in cardiomyocytes from the CIRKO genotype, indicating that they may be associated with the capacity to promote neovascularization, increase vascular diastolic capacity, and maintain mitochondrial polarization during hypoxia. However, the specific mechanisms of the above processes still need to be studied in greater depth (60, 61). The above experiments suggest different influences and mechanisms of action of insulin receptors and related genes in the pathology of DCM and other heart diseases, confirming their important role in the pathology of these heart diseases. **Table 1** briefly describes the role of insulin signaling genes in DCM.

**Table 1:** Insulin signaling transduction genes and their roles in diabetic cardiomyopathy.

Insulin Signaling transduction gene	Main function	Role in diabetic cardiomyopathy	Refs
*IRS1* (Insulin Receptor Substrate 1)	Major regulator of insulin signaling transduction	1. Regulates pancreatic cell function, maintains glucose metabolism and insulin sensitivity.	(65, 66)
		2. Mutations/abnormal expression block PI3K/AKT pathway, causing insulin resistance and diabetes.	(68–70, 72–75, 77)
		3. Deletion of IRS1 & IRS2 affects Akt/FOXO1, reduces ATP supply, promotes myocardial apoptosis, fibrosis, and decline.	(70)
*IRS2* (Insulin Receptor Substrate 2)	Involved in insulin signaling transduction, maintaining normal insulin sensitivity	1. IRS2 knockout leads to diabetes due to insulin resistance and β-cell insufficiency.	(78)
		2. Mutations/abnormal expression cause insulin resistance and metabolic disorders.	(89–93, 96, 97)
		3. Abnormal IRS2 exacerbates myocardial insulin resistance, affecting metabolism and function.	(94, 95)
*PIK3R1* (Phosphatidylinositol 3-Kinase Regulatory Subunit 1)	Essential component of the PI3K pathway, stabilizing and inhibiting p110 catalytic activity	1. Mutations/defects cause insulin resistance and metabolic abnormalities, independent of obesity and dyslipidemia.	(99, 104, 106)
		2. Abnormal PIK3R1 affects PI3K/AKT, impacting myocardial glucose metabolism & function.	(99, 104, 107, 108)
		3. Associated with metabolic disorders like SHORT syndrome.	(105, 106)
*GLUT4* (Glucose Transporter 4)	Insulin-regulated glucose transporter protein responsible for intracellular glucose transport	1. Under insulin stimulation, GLUT4 increases glucose uptake by translocating to the cell membrane.	(113, 114)
		2. Mutations/abnormal expression reduce glucose uptake and cause insulin resistance.	(114, 116, 123)
		3. Abnormal GLUT4 decreases myocardial glucose uptake, affecting metabolism and function.	(114, 117)
		4. Regulated by transcription factors (e.g., MEF2, GEF) and physiological factors (e.g., exercise).	(126–129)

In contrast, the positive promotion of insulin receptors and improvements in the efficiency of insulin signaling in different ways help reduce blood glucose levels. Flavonoids in mulberry leaves can effectively affect the glycolipid metabolism of adipocytes by regulating the IRS1/PI3K/AKT signaling pathway. These flavonoids not only reduce free fatty acid levels and increase glucose consumption but also significantly increase the expression of insulin signaling-related proteins (e.g., phosphorylated IRS1, PI3K, and Akt) in adipocytes and promote the membrane transport of GLUT4 (62). Carvacrolone is a naturally occurring bioactive compound isolated from the ethyl acetate fraction of Bengal banyan lupin leaves. In a recent study, treatment of HepG2 cells with carvacrolone increased the insulin receptor and IRS1 expression, further activating the PI3K/Akt pathway and inhibiting GSK3 and FoxO1 protein synthesis (63). These findings suggest that carvacrolone affects glucose metabolism and insulin signaling through the IRS1/PI3K/Akt/GSK3/FoxO1 pathway. In addition, Qi et al. reported that supplementation with Poria cocos tea attractively increased the expression of IRS1, PI3K, and Akt proteins in the liver in a T2DM mouse model, suggesting that the antidiabetic effect of Poria cocos tea extract (FTE) was mediated by the IRS1/PI3K/Akt signaling pathway (64). These results suggest that targeting the insulin signaling pathway may be a key strategy for controlling glycolipid metabolism in T2DM treatment. Therefore, an in-depth study of the functions and regulatory mechanisms of these genes is important for understanding the pathogenesis of metabolic diseases and developing therapeutic strategies.

### 3.2 Critical insulin signaling genes IRS1, IRS2, PIK3R1 and GLUT4

#### 3.2.1 IRS1

IRS1 and IRS2 in the insulin signaling pathway are dysregulated in diabetes mellitus, so they need to work in concert to maintain normal glucose metabolism and insulin sensitivity (65). Under normal physiological conditions, as a major regulator of pancreatic α-cell function, IRS1 is an insulin receptor substrate and is involved in insulin signaling (65). IRS1 is a major regulator of pancreatic β-cell function (66). In IRS1- or IRS2-knockout mice, the peripheral tissues of the mice exhibit insulin resistance due to the blockage of PI3K/AKT signaling, and the impairment of insulin secretion further contributes to the development of diabetes mellitus (67). The results indicate that IRS1 or IRS2 may facilitate insulin action in the liver, thereby maintaining systemic glucose tolerance, particularly when β-cell function is able to offset insulin resistance. Additionally, the findings imply that IRS1 plays a pivotal role in insulin action in the liver and systemic glucose homeostasis (66).

In a genetic study of a family with early-onset T2DM, scientists identified a rare heterozygous missense mutation (p.His713Tyr) within the IRS1 gene via gene sequencing technology (68). They reported that the p.His713Tyr variant might result in defective binding to PI3K, which leads to an IRS1-associated reduction in PI3K activity and subsequent activation of Akt, ultimately affecting insulin signaling to the cellular carrier pathway and thereby causing diabetes. This study suggested that this gene may be a causative factor for T2DM in this family. In addition, a novel T608R missense mutation in the IRS1 gene was identified in patients with T2DM (69). This IRS1 mutation is defective in its ability to bind and activate PI3K, and as a result, IRS1-T608R has a similarly reduced ability to mediate GLUT4 translocation in adipocytes, a defect that is more prominent in the insulin-free basal state. This mutation has been hypothesized to potentially negatively impact the metabolic transduction of insulin signaling, thereby affecting the course of T2DM development. This finding highlights the important role of specific mutations within the IRS1 gene in regulating the efficiency of insulin signaling, further revealing that these variants may play key roles in the pathogenesis of T2DM. This study revealed a strong association between the IRS1 gene and its surrounding genetic variants and T2DM, insulin resistance, and hyperinsulinemia, emphasizing the central role of IRS1 in the pathogenesis of T2DM. In addition, simultaneous deletion of the IRS1 and IRS2 genes in human cardiomyocytes leads to attenuation of the Akt/FOXO1 signaling pathway, which is accompanied by impaired cardiometabolic gene expression and a reduction in the myocardial availability of ATP, cardiac apoptosis, fibrosis, and cardiac function decline, highlighting the importance of these genes in maintaining cardiac health (70). These findings further emphasize the importance of understanding and regulating the functions of IRS1 and IRS2 in DCM.

In addition, polymorphisms in the IRS1 gene have been found to be associated with an increased risk of developing T2DM. One of the most prevalent polymorphisms is a glycine-to-arginine mutation at codon 972 (G972R) of the IRS1 gene, which is also designated Gly972Arg (71). The Gly972Arg polymorphism is associated with an increased risk of T2DM and insulin resistance. Clinical cohorts indicate that this variant confers a higher risk of cardiovascular disease in females, who exhibit higher metabolic disturbances, suggesting estrogen-mediated compensatory mechanisms (72). In a transgenic mouse model overexpressing the above mutation (Tg972), glucose utilization and insulin signaling in insulin-targeted tissues are impaired, as evidenced by a significant reduction in insulin-stimulated phosphorylation of Tyr 941 and Tyr 989, which reduces IRS1 binding to PI3K. Impaired activation of the insulin pathway leads to reduced tissue-specific insulin action and ultimately systemic insulin resistance as mice age (73). Another study revealed that a variant of G972R in human endothelial cells impaired insulin-stimulated NO release through the IRS1/PI3-K/PDK-1/Akt insulin signaling pathway, leading to endothelial dysfunction and resulting in increased genetic susceptibility to cardiovascular disease (74). However, in a meta-analysis of more than 9,000 individuals, the Gly972Arg polymorphism had no significant effect on various markers of insulin secretion or insulin resistance, and the correlation between the Gly972Arg polymorphism and T2DM was unproven, which may also be related to ethnic differences (75).

The effects of specific cellular transcription factors on IRS1 gene expression have also been reported in recent studies. Transcription factor activator protein 2β (AP-2β) is an important transcription factor that regulates several key genes in the monoamine neurotransmitter system (76). Further studies revealed the overexpression of AP-2β in 3T3-L1 adipocytes, where AP-2β subsequently bound specifically to the IRS1 promoter region, impairing the promoter activity of IRS1 and subsequently decreasing the expression of downstream mRNAs and proteins, which could increase susceptibility to obesity and T2DM (77). Investigating the activating or inhibiting effects of relevant specific cellular transcription factors and cellular protein components on IRS1 and its genes offers potential possibilities for future relevant drug design ideas.

Understanding its role in insulin signaling is crucial for developing new therapeutic approaches for diabetes and metabolic diseases. In addition, studying the mechanisms of this disease in depth can help design drug treatments by enhancing the activity or stability of IRS1, which may help improve insulin sensitivity and provide new ideas for the treatment of T2DM, as well as a deeper understanding of the multiple roles of specific genes, such as IRS1, in physiology, pathology, and drug discovery.

#### 3.2.2 IRS2

Like IRS1, IRS2 plays a key role in maintaining normal insulin sensitivity, and it is a substrate for the insulin receptor involved in insulin signaling. In IRS2 knockout mice, male mice are normal in body size but develop diabetes at approximately 8 weeks of age due to hepatic and peripheral insulin resistance and pancreatic β-cell insufficiency (78). ATF3, an adaptive response gene in the ATF/cAMP response element binding (CREB) family of proteins, is either lowly expressed or not expressed in the normal state and has been shown to be involved in cellular immunity and metabolism in several studies. ATF3 expression is elevated in diabetic cells, suggesting that this proapoptotic gene is upregulated during the pathophysiological development of diabetes. In pancreatic β-cells under inflammatory stress (e.g., cytokine exposure or hyperglycemia), ATF3 directly represses IRS2 transcription by binding to its promoter region (79). This repression is mediated through ATF3’s recruitment of histone deacetylase 1 (HDAC1), which deacetylates histones at the IRS2 locus, leading to chromatin condensation and transcriptional silencing (80, 81). The molecular mechanism involves ATF3 forming a complex with JunB, an AP-1 family transcription factor activated by pro-inflammatory cytokines such as TNF-α and IFN-γ (82). This JunB/ATF3 heterodimer might bind to the CRE/AP-1 motifs within the IRS2 promoter, synergistically suppressing its activity while paradoxically promoting β-cell survival during autoimmune inflammation (83, 84). In an ATF3 knockout mouse model, the absence of this gene prevents HDAC1 recruitment to the IRS2 promoter, resulting in elevated IRS2 expression and enhanced PI3K/Akt signaling (85). Paradoxically, this leads to reduced β-cell apoptosis but exacerbates systemic insulin resistance due to compensatory hyperinsulinemia and β-cell exhaustion (86). Notably, other studies have corroborated the role of ATF3 in IRS2 regulation across tissues. In hepatocytes, ATF3 represses the ChREBP-Scd1 axis, indirectly preserving IRS2 function and glucose homeostasis by reducing lipid-induced insulin resistance (87, 88). Furthermore, ATF3 interacts with the NF-κB p65 subunit in macrophages, suppressing pro-inflammatory pathways that otherwise impair IRS2-mediated insulin signaling through HDAC1-dependent deacetylation of p65 (83, 89, 90).

Similarly, mutations or defects in IRS2 may lead to insulin resistance and metabolic disorders. In certain disease states, such as polycystic ovary syndrome (PCOS), the common GlyAsp polymorphism in IRS2 may be associated with the insulin resistance observed in patients with PCOS. The GlyAsp1057 polymorphism, which is common in IRS2, appears to affect susceptibility to T2DM in both African American and white women with polycystic ovary syndrome, as evidenced by higher glucose concentrations at the 2-hour point in the oral glucose tolerance test (91). Another meta-analysis of the associations between polymorphisms in the IRS1 and IRS2 genes and PCOS revealed that the Gly972Arg polymorphism in IRS1 may increase the risk of PCOS. However, the Gly1057Asp polymorphism of IRS2 was not significantly associated with PCOS, and individuals carrying the IRS1 Arg972 variant presented increased fasting insulin levels and insulin resistance (92). In another study of patients with PCOS and gestational diabetes mellitus, analysis of ovaries by immunoblotting and immunohistochemistry revealed several important changes (93). In polycystic ovaries, IRS1 expression was decreased in the granulosa cells of the follicle and increased in the theca cells of the follicle compared with that in normally ovulating ovaries. This finding suggests that the shift of insulin signaling proteins from IRS1 to IRS2 in follicles may be associated with ovarian abnormalities in PCOS and gestational diabetes mellitus patients (93). The expression of IRS1, IRS2, and Akt-2 was found to be reduced in patients with T2DM, whereas IRS2 plays a key role in regulating human insulin secretion. These findings suggest that the specific expression and function of insulin signaling pathway elements in human pancreatic islet β-cells still need to be further determined (94).

An increase in IRS2 activity or expression can contribute to insulin sensitivity (95). Owing to the role of IRS2 in energy metabolism and lipid metabolism, drugs that target IRS2 may help alleviate obesity and related metabolic diseases (96). More effective regulation of metabolic processes through the modulation of IRS2 activity is needed. Preventing insulin resistance and enhancing IRS2 function may be effective strategies to prevent or treat insulin resistance (97). Given the role of IRS2 in insulin signaling, enhancing its activity could help maintain normal insulin sensitivity and glucose homeostasis.

#### 3.2.3 Phosphatidylinositol 3 kinase (PIK3R1)

PIK3R1 is an important component of the PI3K pathway, forming a complex with the p110 subunit of PI3K. It stabilizes and inhibits p110 catalytic activity, and acts as a connector for interactions with IRS proteins and growth factor receptors, assisting in the activation of Akt-mediated insulin signaling (98). PI3K converts phosphatidylinositol 4,5-bisphosphate (PIP2) to phosphatidylinositol 3,4,5-trisphosphate (PIP3), which recruits the protein kinase AKT to the inner layer of the cell membrane to be activated and involved in various metabolic functions (99). In addition to IRS proteins and AKT, PIK3R1 also interacts with downstream effectors such as PDK1 (3-phosphoinositide-dependent protein kinase-1) and mTORC2, which further phosphorylate and activate AKT, playing a crucial role in glucose uptake and glycogen synthesis (100, 101). Furthermore, PIK3R1 can modulate the activity of GSK3β (glycogen synthase kinase 3 beta), a key regulator of glycogen metabolism, and FOXO transcription factors, which control gluconeogenic gene expression in the liver (99, 102, 103). Through these molecular interactions, PIK3R1 plays a central role in maintaining insulin sensitivity and glucose homeostasis. When PIK3R1 is mutated or defective, it may lead to insulin resistance and metabolic abnormalities independent of obesity and dyslipidemia, due to increased energy expenditure (104). PIK3R1 is a key gene in the insulin signaling pathway, and it works together with other components to regulate glucose metabolism and maintain glucose homeostasis under normal physiological conditions.

PIK3R1 plays an important role in the pathogenesis of systemic metabolic diseases. For example, SHORT syndrome, a rare multisystem disorder, is characterized by short stature, joint hyperextension, ocular pitting, Rieger’s anomaly, and delayed tooth eruption (105). The primary cause of SHORT syndrome is heterozygous loss-of-function mutations in the PIK3R1 gene, which encompass a number of different types of mutations, with Arg649Trp being the most common type. Current research indicates that the disease may be caused by a reduction in the activity of the PI3K-AKT-mTOR pathway (99, 106). In the pathogenesis of T2DM, mutations or malfunctions of the PIK3R1 gene may lead to insulin resistance, which is an important component of T2DM pathogenesis (99). In cardiomyocytes from a mouse model of Pik3r1-/-, insulin-stimulated IRS1- and IRS2-mediated PI3K expression is reduced, and insulin release and insulin sensitivity are decreased. However, PIK3R1 is not a major component of insulin-stimulated PI3K (104). Notably, sex-specific differences in PI3K/Akt pathway activity have been reported, with female rodents demonstrating greater insulin sensitivity in cardiac tissue compared to males, possibly due to estrogen-mediated activation of IRS1 (107–109).

Modulation of PIK3R1 activity holds significant promise for the treatment of diseases, including certain cancers, due to its critical role in the PI3K-AKT signaling pathway (110). PIK3R1 not only stabilizes the p110 catalytic subunit of PI3K but also plays a key role in regulating the phosphorylation of downstream intermediates via Akt activation, which affects multiple physiological processes, including cell growth, metabolism, and survival. Its involvement in cancer is particularly notable, as the dysregulation of the PI3K pathway is a common feature in various cancers. Recent studies have explored PIK3R1 as a therapeutic target in the context of breast cancer, prostate cancer, and glioblastoma, where its inhibition has shown promise in preclinical models by reducing tumor growth and enhancing the effects of chemotherapy and targeted therapies (111). Furthermore, modulating PIK3R1 activity may also improve insulin sensitivity and metabolic regulation, particularly in metabolic disorders like T2DM (112). Although the research on PIK3R1 as a therapeutic target is still in its early stages, understanding its precise mechanisms of action could pave the way for the development of novel drugs aimed at improving glucose metabolism and potentially addressing insulin resistance. Further investigation into these mechanisms is crucial for optimizing therapeutic strategies that could harness PIK3R1 modulation for disease management.

#### 3.2.4 GLUT4

GLUT4 is an insulin-regulated glucose transporter protein found in large amounts in intracellular vesicles in adipose and muscle cells under low-insulin conditions. Upon insulin stimulation, activation of the IRS/PI3K/Akt signaling axis facilitates GLUT4 translocation to the plasma membrane, enhancing glucose uptake (113). In T2DM patients, impaired GLUT4 trafficking reduces membrane GLUT4 density, which not only diminishes glucose influx but also exacerbates systemic insulin resistance through multifaceted mechanisms (114). First, diminished glucose uptake forces cardiomyocytes to rely predominantly on FAO for ATP production (115). However, excessive FAO could inhibit IRS1 tyrosine phosphorylation and attenuate PI3K/Akt signaling, establishing a self-reinforcing cycle of insulin resistance (29, 116). Second, mitochondrial dysfunction arising from impaired glucose utilization leads to electron transport chain inefficiency and elevated ROS production (117). ROS oxidatively modifies SH2 domains of IRS1/2, disrupting their interaction with insulin receptors and further compromising the signaling cascade required for GLUT4 translocation (118–120). Additionally, chronic hyperglycemia promotes AGE-mediated activation of the RAGE receptor, triggering NF-κB inflammatory pathways (121). This upregulates pro-inflammatory cytokines like TNF-α, which destabilize GLUT4 mRNA and suppress its transcriptional expression (122).

It is important to notice that the regulation of GLUT4 gene expression is influenced by various factors. For example, a study revealed that the overexpression of cytochrome P450 isoform 2E1 (CYP2E1) in skeletal muscle L6 and primary rat adipose (PRA) cells repressed the gene at both the promoter and mRNA levels. In contrast, silencing CYP2E1 significantly increased the endogenous mRNA and protein levels of the transporter. Additionally, CYP2E1 overexpression in L6 cells inhibited insulin-stimulated translocation of the protein to the cell surface, impairing glucose uptake (123). In addition, exercise increases GLUT4 mRNA transcription in the skeletal muscle of T2DM patients, promoting GLUT4 production and attenuating impaired glucose uptake (124). However, these increases are transient, returning to pre-exercise levels within 18–24 hours. The prolonged increase in the transporter protein level induced by exercise training is believed to result from the cumulative effect of these transient increases in its mRNA level, which stimulate protein synthesis over the long term (125).

Transgenic studies have identified two conserved regions on the promoter in human skeletal muscle essential for normal expression: the myocyte enhancer factor 2 (MEF2) transcription factor and the GLUT4 enhancer factor (GEF), both of which are crucial for proper GLUT4 expression in skeletal muscle (126). A study analyzing the DNA-binding activity of MEF2 and GEF after 60 minutes of cycling revealed that exercise increased their binding activity by 1.6-fold and 1.4-fold, respectively (127). These results indicate that MEF2 and GEF DNA-binding activities are not the same and suggest that they play important roles in mediating GLUT4 production. Identifying the upstream regulators of these transcription factors may be critical for understanding the regulation of GLUT4.

Several studies have indicated that exercise promotes an increase in GLUT4 protein, but whether exercise clearly improves insulin resistance and glucose regulation is not yet fully understood, and relevant experiments could be refined in future studies to demonstrate its relevance. Second, in studies of GLUT4 gene polymorphisms, researchers have evaluated the frequencies of GLUT1 and GLUT4 alleles and genotypes in Asian, Caucasian, and African American controls and diabetic patients. The data revealed that GLUT4 polymorphisms were not associated with T2DM in any of the populations studied, and the allele frequencies of GLUT4 did not differ between males and females or between subjects with BMIs below or above 24/25, suggesting that the current GLUT4 gene is not associated with T2DM (127).

**Figure 3:**
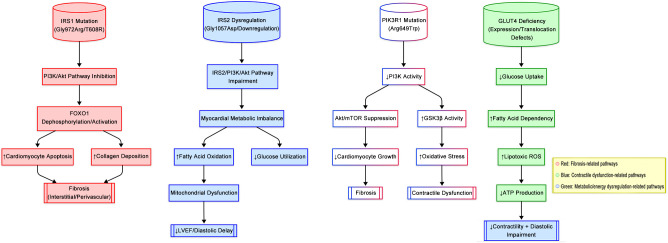
Genetic alterations in insulin signaling genes drive distinct pathophenotypes of DCM.

Interestingly, in one study, the use of peripheral muscle electrical stimulation increased GLUT4 levels, improved morphological changes in the skeletal muscle of rats with HF, and ameliorated HF (128, 129). However, the exact mechanism is not clear. Recent experiments suggest that Shengxian Tang can improve cardiac function and ventricular remodeling, accompanied by the restoration of GLUT4 content in cardiomyocytes, facilitation of mitochondrial glucose utilization, and amelioration of glucose metabolism disturbances in combination with systolic dysfunction (129). Multiple experiments have demonstrated that enhancing the intracellular GLUT4 content and promoting its translocation contribute to improving peripheral glucose utilization and provide new ideas for the treatment of T2DM, with the potential to explore GLUT4 as a therapeutic target. According to the previous studies, the effects of mutations in IRS1, IRS2, PIK3R1, and GLUT4 on DCM pathogenesis are illustrated in **Figure 3**.

### 3.3 Epigenetic mechanisms in diabetic cardiomyopathy

#### 3.3.1 DNA methylation and metabolic memory

DNA methylation alterations in key metabolic genes contribute to persistent metabolic dysfunction in DCM. For instance, maternal hyperglycemia induces hypomethylation of the TET3 gene in oocytes, leading to impaired oxidative demethylation of paternal DNA in offspring (130). This results in hypermethylation of insulin secretion-related genes (e.g., Gck) in pancreatic islets, causing glucose intolerance and insulin resistance in subsequent generations (130). Such “metabolic memory” explains why glycemic control may not fully reverse diabetic complications once established.

#### 3.3.2 Histone modifications and oxidative stress

The Nrf2-ARE pathway, a key antioxidant defense system, is epigenetically regulated by histone acetylation (131). Hyperglycemia reduces histone acetyltransferase activity (e.g., p300/CBP) and increases histone deacetylase (HDAC) expression, suppressing Nrf2-driven transcription of antioxidant enzymes (e.g., HO-1) (132, 133). This exacerbates oxidative stress and mitochondrial dysfunction in cardiomyocytes. Conversely, SIRT3 (a class III HDAC) activation mitigates oxidative damage by deacetylating mitochondrial proteins, suggesting therapeutic potential (134).

#### 3.3.3 Non-coding RNAs and insulin signaling

MicroRNAs (miRNAs) and long non-coding RNAs (lncRNAs) exert profound regulatory effects on insulin signaling pathways in DCM through transcriptional and post-transcriptional mechanisms. MiR-133a, a cardioprotective miRNA, is downregulated under hyperglycemic conditions, leading to direct suppression of GLUT4 mRNA translation and impaired glucose uptake in cardiomyocytes (135). This defect exacerbates myocardial energy deficiency and contractile dysfunction in diabetic hearts (136). Concurrently, miR-29c contributes to myocardial fibrosis by targeting PI3K and Akt, disrupting insulin-mediated anti-fibrotic signaling and promoting diastolic dysfunction (137, 138). Beyond miRNAs, lncRNAs such as KCNQ1OT1 play a chromatin remodeling role by recruiting Polycomb repressive complexes to the promoter region of IRS1, thereby silencing its transcription and amplifying systemic insulin resistance (139, 140). This epigenetic dysregulation establishes a feedforward loop that perpetuates metabolic derangements in DCM.

#### 3.3.4 Novel protein post-translational modifications

Emerging evidence highlights the critical role of metabolite-driven post-translational modifications (PTMs) in modulating cardiac energy metabolism and insulin sensitivity (141). Hyperglycemia-induced lactate accumulation drives histone H3K18 lactylation (Kla), a glucose-dependent modification that activates transcription of pro-hypertrophic genes (e.g., ANP and BNP), thereby exacerbating pathological ventricular remodeling (142). In contrast, β-hydroxybutyrate-mediated histone H3K9 β-hydroxybutyrylation (Kbhb) enhances VEGF expression under ketotic conditions, which may transiently improve endothelial function (143). However, dysregulated Kbhb promotes lipid deposition in cardiomyocytes, creating a paradoxical balance between vascular protection and lipotoxicity (144). Mitochondrial dysfunction in diabetic hearts is further aggravated by succinyl-CoA accumulation, which induces succinylation (Ksucc) of CPT2, a key enzyme in fatty acid oxidation (145, 146). This modification impairs mitochondrial β-oxidation capacity, leading to lipid droplet accumulation and oxidative stress. Notably, SIRT5-mediated desuccinylation has emerged as a potential therapeutic target, as its activation restores CPT2 activity and alleviates cardiac lipotoxicity (147). These PTMs collectively contribute to the “metabolic memory” phenomenon in DCM, highlighting their dual roles as both pathological drivers and potential intervention points.

## 4 Treatment of DCM

### 4.1 Translational implications of insulin signaling genes

While the molecular intricacies of insulin signaling genes in DCM pathogenesis are well-delineated, their translational potential remains underexplored. Emerging evidence underscores the feasibility of leveraging genetic insights to refine diagnostic paradigms and therapeutic strategies. For instance, polymorphisms in IRS1 (e.g., Gly972Arg) and IRS2 (e.g., Gly1057Asp) are not merely mechanistic curiosities but clinically actionable biomarkers (148, 149). Population-scale genome-wide association studies (GWAS) have identified these variants as predictors of accelerated cardiac remodeling in diabetic cohorts, suggesting their utility in risk stratification for early-stage DCM (149). Integrating such genetic markers with conventional diagnostic tools (e.g., echocardiography, serum BNP levels) could enable a precision medicine approach to identify high-risk patients warranting aggressive intervention (150).

Therapeutically, GLUT4 expression dynamics offer a paradigm for targeted drug development. Studies suggest that aloperine ameliorates diabetic metabolic disorders (e.g., hyperglycemia, insulin resistance, lipotoxicity) and inflammatory responses through multi-target mechanisms, which may indirectly prevent or delay the onset and progression of diabetic heart disease (151). However, its direct mechanisms of action on cardiomyocytes (e.g., the ERK1/β-catenin pathway) still require further validation in diabetic cardiomyopathy models. Similarly, PIK3R1 mutations associated with SHORT syndrome present a unique opportunity for repurposing PI3K inhibitors to ameliorate insulin resistance in DCM (152–154). Preclinical studies have demonstrated that alpelisib, a selective PI3Kα inhibitor, restores insulin sensitivity in murine models harboring PIK3R1 mutations (155). Intriguingly, emerging evidence reveals a paradoxical therapeutic role for PI3Kα pathway modulation in DCM (156). While pharmacological inhibition of PI3Kα may address insulin resistance, targeted overactivation of this pathway *in vivo* has been shown to ameliorate key pathological hallmarks of DCM, including cardiomyocyte apoptosis, interstitial fibrosis, maladaptive cardiac hypertrophy, and diastolic dysfunction (157). This dichotomy implies that tissue-specific targeting of PI3Kα, which balances systemic metabolic advantages with direct cardioprotective effects, could potentially revolutionize the management of diabetic cardiovascular complications.

Despite the therapeutic potential of targeting insulin signaling genes in DCM, translational challenges persist due to the pleiotropic effects of these molecular regulators. For example, while IRS1 activation augments glucose metabolism, its overexpression paradoxically exacerbates hypertrophic signaling via mTOR hyperactivation (49, 158). Recent advances in nanotechnology offer promising solutions to this conundrum. As exemplified by Wang et al., nanoparticle-mediated delivery of TLR4 siRNA selectively attenuated myocardial inflammation and fibrosis in diabetic rats without disrupting systemic glucose homeostasis, demonstrating the feasibility of cardiac-targeted gene silencing (159). Building upon this paradigm, nanoparticle-encapsulated IRS1 siRNA could theoretically achieve localized suppression of IRS1-driven mTOR overactivation and ameliorate cardiac fibrosis while preserving its glucoregulatory functions. Although direct evidence in DCM remains unreported, the synergistic combination of tissue-specific siRNA delivery and pathway-selective gene modulation presents a compelling framework for overcoming the pleiotropy barrier in insulin signaling therapeutics.

### 4.2 Traditional pharmacotherapy

DCM is a unique condition, and therapeutic drugs may have different effects on cardiovascular endpoint events in diabetic patients. Patients with diabetes are at markedly elevated risk of developing cardiovascular disease. Effective glycemic control can mitigate the likelihood of cardiovascular incidents. Past epidemiological evidence suggests that among diabetic patients with a glycated hemoglobin (HbA1c) greater than or equal to 7.0%, each 1% increase in HbA1c results in an 8% increase in the risk of developing atrial fibrillation, and each 1% decrease in HbA1c reduces the risk of diabetes-related cardiovascular disease and mortality by 21% (160, 161). It can be reasonably assumed that effective improvement of average blood glucose levels in patients who are controlled with medications would result in a reduction in the incidence of diabetic cardiomyopathy. The question thus arises as to whether it is preferable to maintain HbA1c levels at their lowest possible level. Nevertheless, a U-shaped relationship has been identified between HbA1c and mortality in diabetic patients with HF. Patients with moderate glycemic control (7.1% < HbA1c ≤ 7.8%) have been found to have the lowest risk of death (162). Therefore, improving patient prognosis by solely reducing blood glucose levels is challenging. Moreover, it is hypothesized that hypoglycemia may have a deleterious impact on the cardiovascular system via the stimulation of sympathetic nerves and the subsequent inflammatory response (163). Consequently, the avoidance of hypoglycemia is crucial to consider. And more, a meta-analysis of 37,229 patients revealed that intensive glycemic control in patients with T2DM did not reduce the risk of hospitalization for HF (164). Another meta-analysis of 13 studies comprising 34,533 patients with T2DM demonstrated that intensive glucose-lowering therapy did not reduce the incidence of cardiovascular events but was observed to increase the risk of HF by 47%. The potential benefits of intensified hypoglycemic therapy may be offset by the hazards associated with severe hypoglycemia (165).

Diabetes treatment is based on diet and exercise. However, exercise in DCM also promotes myocardial mitochondrial biogenesis, autophagy, fusion and division balance, improves oxidative stress, and enhances myocardial mitochondrial metabolism through signaling pathways such as PGC-1α, SIRT3, and FGF21, thus resisting DCM (166). Furthermore, studies conducted on healthy individuals have demonstrated that limiting caloric intake by 15% for a period of two years can lead to improvements in health-related quality of life and a reduction in oxidative stress compared to individuals who did not implement such dietary restrictions. However, the efficacy of calorie restriction in the treatment of HF in humans remains to be established (167).

The current study demonstrated that different classes of glucose-lowering medications exert disparate effects on the incidence of cardiovascular events. A retrospective study comparing the risk of myocardial infarction and total mortality revealed that, in comparison with metformin monotherapy, sulfonylureas may potentially elevate the risk of developing HF, with an 18–30% increase in HF with sulfonylureas alone (168, 169). Insulin has the effect of reducing myocardial oxygen consumption and increasing cardiac efficiency by increasing the proportion of glucose used as a source of energy for the heart. In addition, insulin promotes contraction of cardiomyocytes while positively affecting myocardial diastole. It enhances ribosome biosynthesis and protein synthesis and stimulates vascular endothelial growth factor, thereby promoting angiogenesis, cell survival, and the inhibition of apoptosis. This ultimately improves myocardial microcirculation and coronary arterial resistance, leading to increased myocardial blood perfusion. Thus, insulin exerts a direct effect on the myocardium, which is primarily mediated through the PKB/Akt signaling pathway (170). Pioglitazone, a thiazolidinedione, increases the risk of HF by activating sodium transporters in the proximal renal tubule and epithelial sodium channels in the collecting ducts via peroxisome proliferator-activated receptor gamma (PPARγ), which encourages the reabsorption of Na+, resulting in fluid retention (171). Therefore, we recommend combining these drugs with thiazide diuretics or mineralocorticoid receptor antagonists to prevent and treat HF due to fluid retention.

The efficacy of DPP4 inhibitors in the treatment of HF remains a topic of debate. According to recent reports, DPP4 inhibitors have been reported to have no discernible effect on left ventricular diastolic function in diabetic patients compared with a control group (172). In some long-term cardiovascular safety studies, DPP4 inhibitors did not result in a statistically significant increase in the incidence of heart failure compared to the use of a placebo (173, 174). However, saxagliptin does not increase or decrease the incidence of cardiac ischemic events, but there is an increased incidence of hospitalization for HF (175). It is possible that DPP4 inhibitors increase the risk of HF by increasing sympathetic activity and the levels of bioactive proteins in the body. However, further research is needed to confirm whether there is a causal relationship between DPP4 inhibitors and HF (176).

Recent guidelines for the diagnosis and treatment of acute and chronic HF published by the European Society of Cardiology (ESC) state that, compared with insulin and sulphonylureas, metformin is considered safe for patients with HF (177). Metformin activates the enzyme AMP-activated protein kinase (AMPK), which regulates energy metabolism in a variety of tissues, including the heart, liver, and muscle. The administration of metformin has been demonstrated to enhance left ventricular function and extend survival in murine models of ischemia-induced hypertension (178). In a dog model of HF, metformin attenuated oxidative stress-induced cardiomyocyte apoptosis, activated AMPK, and inhibited the progression of HF. As evidenced by the latest randomized controlled trials and meta-analyses, metformin has been validated to confer significant benefits in DCM, particularly among patients with T2DM concurrent with congestive HF. Metformin can reduce the risks of all-cause mortality and cardiovascular-related mortality in these patients, while enhancing cardiac function without elevating the risk of hospitalization due to HF (179, 180). Consequently, the efficacy and safety of metformin in the treatment of HF patients with T2DM have been firmly established. The interplay between insulin signaling and AMPK/mTOR pathways is critical in DCM pathogenesis. AMPK activation by metformin counteracts insulin resistance via enhancing GLUT4 translocation, while mTOR overactivation in diabetic hearts exacerbates fibrosis (181). Targeting these crosstalk mechanisms may offer novel therapeutic avenues.

SGLT2 inhibitors constitute a new class of hypoglycemic agents used to treat T2DM. The mechanism of action of these drugs is to inhibit glucose reabsorption in the early proximal renal tubules, thereby promoting urinary glucose excretion, reducing the body’s glucose burden, and reducing the circulatory burden (182). The SGLT2 inhibitor canagliflozin has also been shown to reduce the number of HF hospitalizations and cardiovascular event mortality in T2DM mellitus patients at high cardiovascular risk in controlled clinical trials (183). It is anticipated that SGLT2 inhibitors will have a broad preventive impact on HF in patients with diabetes. SGLT2 inhibitors exert a preventative and ameliorative effect on HF through hemodynamic mechanisms and metabolic processes (184). Hemodynamic mechanisms encompass diuretic effects and the regulation of blood volume. Metabolic mechanisms include hypoglycemic effects, prevention of lipotoxicity, improvement of insulin resistance, weight loss, reversal of ventricular remodeling, and optimization of cardiac energy metabolism, which together prevent and improve DCM-induced HF (185). Recent large-scale clinical trials further validate the therapeutic potential of SGLT2 inhibitors in HFpEF. The EMPEROR-Preserved trial demonstrated that empagliflozin significantly reduced the composite risk of cardiovascular death or heart failure hospitalization by 21% in 5,988 HFpEF patients (LVEF >40%), primarily driven by a 29% reduction in heart failure hospitalizations (186). Notably, this benefit was consistent across predefined subgroups, including patients with and without diabetes, highlighting its clinical relevance beyond glycemic control.

It is noteworthy that, as indicated by recent systematic review and meta-analysis of 21,947 patients (35.7% female) from 5 randomized controlled trials (RCTs), SGLT2 inhibitors have demonstrated a remarkable capacity to mitigate major adverse outcomes in both male and female patients with HF (187). However, a notable sex difference exists in their therapeutic effects. When compared with the placebo group, both male and female patients exhibited a comparable magnitude of risk reduction in terms of major outcomes. Nevertheless, a closer examination of the data reveals a more nuanced picture. Women were found to have a 32% higher odds ratio of experiencing major outcomes compared to their male counterparts (187). This finding implies that, despite the similar risk ratio reductions observed in both genders, the clinical benefit derived from SGLT2 inhibitors may be attenuated in women. Further research is warranted to elucidate the underlying mechanisms responsible for this disparity and to develop sex-specific treatment strategies.

### 4.3 Nanotherapy

In addition to the above treatments, nanotherapeutics, which have attracted a lot of attention in recent years, have also been applied to the treatment of DCM. In DCM patients, persistent oxidative stress aggravates disease progression. Therefore, Zhang et al. used PEG-modified nanoliposomes (PEG-lips) loaded with non-dense acidic fibroblast growth factor (NM-aFGF) preparation (NM-aFGF-PEG-lips), combined with ultrasound-targeted microbubble disruption technique for increased drug delivery (188). This strategy specifically targets myocardial tissue, with NM-aFGF exerting an inhibitory effect on myocardial oxidative stress and overall improvement of myocardial structural and functional lesions. Due to disruption of macrophage polarization type (M1/M2) can also lead to disease exacerbation. Based on this, Jia et al. covalently coupled sulfhydryl-modified antagonist-miR155 with gold nanoparticles (AuNP) to prepare miR155-AuNP (189). The nanoparticles significantly increased the M2 ratio, thereby reducing the inflammatory effect and significantly alleviating disease symptoms. In addition, the increased ratio of free fatty acid oxidation in patients also affects cardiac efficiency. Ji et al. prepared APS-nano using astragalus polysaccharide (APS) with liposomal nanoparticles (190). The nanoparticles could regulate fatty acid translocase (FAT)/CD36 expression, reduce total cholesterol, triglyceride, and free fatty acid content, regulate lipid metabolism, and improve cardiac metabolism. Zhang et al. prepared PLGA-GLP-1 nanoparticles using poly (ethylene glycol) glycine as a drug carrier (191). It similarly showed a great protective effect on myocardial tissue by promoting mitochondrial DNA repair in cardiomyocytes.

Given that coronary microvascular dysfunction affects the onset and progression of DCM, Mao et al. prepared propylene glycol sodium sulfate (PSS) nanoparticles loaded with alginate (PSS-NP) (192). PSS-NP has the effect of improving cardiac function and restoring myocardial morphology, in addition to increasing the number of microvessels and regulating the function of coronary microcirculation, which is another new method for treating coronary microcirculation dysfunction in DCM. Compared with treatment targeting a single factor, performing multifactorial combination therapy is more effective for DCM treatment. For example, SeNPs can reduce cardiomyocyte apoptosis and promote mitochondrial DNA repair by improving the antioxidant capacity and also inhibit the NF-κB signaling pathway and reduce the inflammatory response (193). Silver nanoparticles (SmSNPs) derived from Syzygium cumini seeds improved antioxidant capacity and attenuated cardiomyocyte damage in diabetic mice (194).

Despite the therapeutic promise of nanomedicines in DCM, several translational challenges must be addressed to advance these innovations into clinical practice. First, biodistribution limitations hinder cardiac-specific delivery. For example, gold nanoparticles (e.g., miR155-AuNP) and PEGylated liposomes (e.g., NM-aFGF-PEG-lips) exhibit nonspecific accumulation in the liver and spleen, reducing their efficacy in targeting myocardial tissue (195–197). Strategies such as surface modification with cardiac homing peptides (e.g., atrial natriuretic peptide analogs) or antibody-conjugated nanoparticles could enhance cardiac specificity (198, 199). Second, immunogenicity concerns pose risks for chronic administration. Anti-PEG antibodies induced by PEGylated formulations (e.g., PLGA-GLP-1) may accelerate blood clearance and compromise therapeutic effects (200). Alternatives like zwitterionic polymers or biodegradable lipid nanoparticles could mitigate immune recognition (201, 202). Additionally, metal-based nanoparticles (e.g., Zn NPs) require rigorous evaluation of dose-dependent cytotoxicity and long-term organ retention (203). Third, regulatory and manufacturing hurdles delay clinical translation. Scalable synthesis under Good Manufacturing Practice (GMP) standards remains elusive for complex formulations such as polysaccharide sulfate-loaded PLGA nanoparticles (204). Batch-to-batch variability in polymer degradation kinetics (e.g., PLGA carriers) further complicates reproducibility (205). Collaborative efforts among academia, industry, and regulatory bodies are essential to establish standardized protocols for nanoparticle characterization and safety assessment.

To sum up, nanomedicines are poised to continue playing a pivotal role in the therapeutic management of DCM owing to their excellent targeting capabilities and therapeutic efficacy. Moreover, integrating nanotherapeutics with insulin signaling gene therapeutics, utilizing insulin signaling genes as therapeutic targets, represents a promising avenue of research for the treatment of DCM. **Table 2** presents an overview of nanomedicines designed to reduce myocardial injury.

**Table 2:** Nanomedicines to reduce myocardial damage.

Mechanism	Shapes	Nanoparticles	Avenues	Refs
Reducing myocardial damage	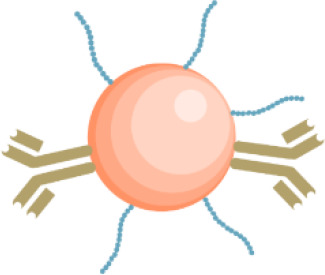	CHP-SPIO-ab MMP2	Specific targeting of cardiomyopathy and matrix metalloproteinase 2 (MMP2) for early fibrosis detection	(206)
		aFGF-NP	Reduces myocardial fibrosis and apoptosis, while increasing myocardial microvessel density.	(207)
		bFGF-lip	Reduced cardiomyocyte apoptosis and increased microvessel density through activation of the PI3K/AKT signaling pathway.	(208)
		NM-aFGF-PEG-lips	Non-mitotic acidic fibroblast growth factor (NM-aFGF) inhibits myocardial oxidative stress injury and ameliorates myocardial structural and functional damage in diabetic cardiomyopathy (DCM) through activation of the AKT/GSK/Nrf-2 signaling pathway.	(188)
		PLGA-GLP-1	Protection against myocardial injury by reducing cardiomyocyte apoptosis and promoting mitochondrial DNA repair in cardiomyocytes.	(191)
Reducing myocardial damage	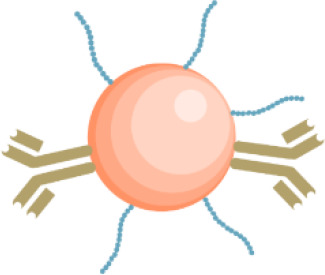	CaPs	Improvement of myocardial contractility.	(209)
		Zn NPs	Improves antioxidant capacity, reduces cardiomyocyte apoptosis and promotes mitochondrial DNA repair.	(210)
		bFGF-NP	Inhibition of myocardial oxidative stress injury and activation of AKT/GSK/Nrf-2 signaling pathway to ameliorate myocardial structural and functional injury.	(192)
Anti-inflammatory and antioxidant	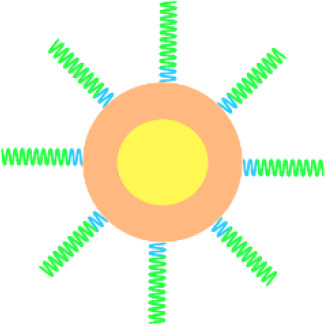	miR155-AuNP	Modulation of M1/M2-type macrophage polarization, reduction of inflammation and apoptosis and restoration of cardiac function.	(189)
Microcirculatory disorder	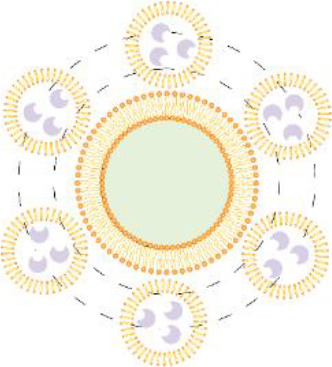	PSS-NP	Improvement of myocardial morphology and structural disorders of coronary microcirculation, increasing the number of microvessels.	(192)
		Polysaccharide Sulfate-Loaded Poly (lactic-co-glycolic acid) Nanoparticles	Improvement of cardiac function and myocardial morphology and increase in the number of microvessels with inhibition of the NF-κB signaling pathway.	(211)

Additionally, to address the heterogeneity of therapeutic strategies for DCM, we present a structured comparison of conventional and emerging therapies (**Table 3**). While SGLT2 inhibitors and metformin demonstrate robust clinical benefits in improving cardiac metabolism and reducing HF hospitalization, their pleiotropic effects limit broader utility. In contrast, emerging nanotherapeutics exhibit superior mechanistic specificity by targeting oxidative stress or insulin signaling pathways, yet require clinical validation. Future efforts should prioritize tissue-selective delivery systems and combinatorial approaches to optimize efficacy while minimizing systemic toxicity.

**Table 3:** Comparison of conventional and emerging therapies for DCM: mechanisms, cardiac function parameters, and clinical evidence.

Therapy category	Treatment	Mechanism specificity	Effect on cardiac function parameters	Clinical trial outcomes	Refs
Traditional Therapy	Diet and Exercise	Improve overall health through caloric restriction and increased physical activity	Exercise promotes cardiac mitochondrial biogenesis, autophagy, fusion, and fission balance, improves oxidative stress, and enhances cardiac mitochondrial metabolism	Exercise demonstrates benefits in improving health-related quality of life and reducing oxidative stress in healthy individuals, but its efficacy in HF treatment is not fully established	(166, 167)
			Caloric restriction reduces oxidative stress and improves quality of life		(168, 169)
	Antidiabetic Drugs (Sulfonylureas)	Lower blood glucose levels through various mechanisms	May increase the risk of HF	Multiple studies suggest a potential increase in HF risk	(170)
	Antidiabetic Drugs (Insulin)	Increases the proportion of glucose used as cardiac energy, reduces myocardial oxygen consumption, enhances cardiac efficiency, and promotes myocardial contraction and relaxation	Reduces myocardial oxygen consumption, increases cardiac efficiency, promotes myocardial contraction and relaxation, and improves myocardial microcirculation	Shows improved cardiac function in animal models	(171)
	Antidiabetic Drugs (Pioglitazone)	Activates PPARγ receptors, increasing sodium transport	Increases sodium reabsorption via PPARγ activation, leading to fluid retention and increased HF risk	Known to increase HF risk, recommended for use in combination with diuretics or mineralocorticoid receptor antagonists	(172–175)
	Antidiabetic Drugs (DPP-4 Inhibitors)	Controversial effects on left ventricular diastolic function	Controversial effects on left ventricular diastolic function	Some studies show no significant impact on cardiac function, but saxagliptin trials demonstrate increased HF hospitalization rates	(178–180)
			No significant increase in HF incidence in long-term cardiovascular safety studies, but certain drugs (e.g., saxagliptin) may increase HF hospitalization rates		(182–186)
	Antidiabetic Drugs (Metformin)	Activates AMPK, enhances GLUT4 translocation	Activates AMPK, enhances left ventricular function, prolongs survival, reduces all-cause and cardiovascular-related mortality, improves cardiac function without increasing HF hospitalization risk	Demonstrates significant benefits in HF patients with T2DM in multiple randomized controlled trials and meta-analyses	(188)
	Antidiabetic Drugs (SGLT2 Inhibitors)	Inhibits early glucose reabsorption in the renal proximal tubule, promoting urinary glucose excretion, and reducing glucose and circulatory burden	Inhibits renal glucose reabsorption, reduces glucose burden, and decreases HF hospitalizations and cardiovascular event mortality	Reduces HF hospitalizations and cardiovascular event mortality in T2DM patients at high cardiovascular risk	(189)
Emerging Therapy	Nanotherapy (NM-aFGF-PEG-lips)	Utilizes nanoparticles as drug delivery carriers to enhance drug solubility, bioavailability, and reduce toxicity and immunogenicity	Inhibits myocardial oxidative stress injury and improves myocardial structural and functional damage	Demonstrates significant therapeutic effects in animal models but not yet widely applied in human clinical trials	(190)
	Nanotherapy (miR155-AuNP)	Prepared by covalently binding thiol-modified miR155 antagonist to AuNP, significantly increasing the proportion of M2 macrophages and reducing inflammatory responses	Regulates M1/M2 macrophage polarization, reduces inflammation and apoptosis, and restores cardiac function	Significantly increases M2 macrophage proportion, reduces inflammatory response, and alleviates disease symptoms	(191)
	Nanotherapy (APS-nano)	Prepared using APS with liposomal nanoparticles to regulate FAT/CD36 expression and lipid metabolism	Enhances cardiac metabolism, potentially indirectly improving cardiac function through lipid metabolism regulation	Regulates lipid metabolism and improves cardiac metabolism	(192)
	Nanotherapy (PLGA-GLP-1)	Prepared using PLGA as a drug carrier to produce PLGA-GLP-1 nanoparticles, promoting mitochondrial DNA repair in cardiomyocytes	Promotes mitochondrial DNA repair in cardiac myocytes and protects myocardial tissue	Demonstrates significant protective effects on myocardial tissue	(193, 194)
	Nanotherapy (PSS-NP)	Improves cardiac function, restores myocardial morphology, and increases microvascular density	Enhances cardiac function, restores myocardial morphology, and increases microvascular density, directly contributing to improved cardiac function	Improves cardiac function, restores myocardial morphology, increases microvascular density, and regulates coronary microcirculation	(166, 167)
	Nanotherapy (SeNPs and SmSNPs)	SeNPs reduce cardiomyocyte apoptosis by enhancing antioxidant capacity; SmSNPs improve antioxidant capacity and alleviate cardiomyocyte injury	Exhibits protective effects on diabetic mice by enhancing antioxidant capacity and reducing myocardial cell injury	Shows protective effects on diabetic mice	(168, 169)

T2DM, type 2 diabetes mellitus; HF, heart failure; FAT, fatty acid translocase.

## 5 Conclusion and prospect

This review marks a significant step forward in our understanding of the intricate mechanisms and therapeutic targets involving insulin signaling transduction genes in DCM. By thoroughly examining the pivotal roles of key genes such as IRS1, IRS2, PIK3R1, and GLUT4, we have elucidated their critical contributions to insulin signaling pathways, glucose metabolism, and myocardial function. Our in-depth analysis has emphasized the central importance of insulin resistance and metabolic disturbances in the pathogenesis of DCM, shedding light on the complex interplay between insulin signaling genes and cardiac dysfunction.

Beyond summarizing existing knowledge, this paper has made a meaningful contribution to the treatment landscape of DCM by proposing insulin signaling genes as viable therapeutic targets. Despite the therapeutic promise of nanomedicines, their clinical implementation is hindered by unresolved challenges. Biodistribution inefficiencies, immunogenic risks, and regulatory complexities necessitate multidisciplinary collaboration to optimize nanoparticle design and validate safety in human trials. Future research should integrate pharmacokinetic-pharmacodynamic modeling and leverage advances in biomaterials to overcome these barriers. Addressing these issues will be pivotal for translating preclinical success into clinically viable therapies for DCM.

While we fully acknowledge that much more research is needed to fully unravel the complexities of DCM and develop effective treatment strategies, this paper has provided novel insights and directions that lay the groundwork for future investigations. By highlighting the potential of targeting insulin signaling genes and exploring advanced nanomedicines, we have added a valuable piece to the literature, paving the way for transformative advancements in the treatment of DCM. Furthermore, we urge future studies to explore the impact of lifestyle modifications, such as diet and exercise, on the expression of insulin signaling genes in DCM patients, as these may offer additional therapeutic avenues.
